# Inflexible habitual decision-making during choice between cocaine and a nondrug alternative

**DOI:** 10.1038/s41398-019-0445-2

**Published:** 2019-03-06

**Authors:** Youna Vandaele, Caroline Vouillac-Mendoza, Serge H. Ahmed

**Affiliations:** 1 Department of Psychological and Brain Sciences, Krieger School of Arts and Sciences, Johns Hopkins University, Baltimore, Maryland 21218 USA; 2 Université de Bordeaux, Institut des Maladies Neurodégénératives, UMR 5293, 146 rue Léo-Saignat, F-33000 Bordeaux, France; 3 CNRS, Institut des Maladies Neurodégénératives, UMR 5293, 146 rue Léo-Saignat, F-33000 Bordeaux, France

## Abstract

The concept of compulsive cocaine-seeking habits is difficult to reconcile with other evidence showing that humans and even rats remain able to shift their choice away from the drug and toward an alternative nondrug reward, when available. This paradox could dissolve if preference for the nondrug option reflected in fact inflexible habitual decision-making (i.e., fixed in a habitual control mode, with no return to a goal-directed control mode). Previous research in rats has shown that prior drug use can favor habit formation, but whether the resulting habits are inflexible or not is largely unknown. Here we addressed this question by manipulating the value of water in rats that chose between water and cocaine in a discrete-trials procedure. Rats preferred water when thirsty and maintained this preference despite water devaluation by satiation. Only with repeated daily testing under water satiation did they progressively reverse their preference toward cocaine. Additional evidence showed that this progressive reversal of preference reflected in fact new interoceptive discrimination learning. Overall, this study suggests that rats seem to be stuck in a habitual decision-making mode, unable to return to a goal-directed mode upon experiencing a change in options value. It also reveals that inflexible decision-making does not necessarily promote drug choice, but can also under some circumstances favor abstinence.

## Introduction

It has long been hypothesized that addictive behaviors emerge from the progressive development and dominance of drug habits over goal-directed control^1,2^. In this framework, drug seeking becomes habitual through repeated consumption and association with environmental context and stimuli and, as a result, is no longer mediated by the expected intoxicating effects of the drug. Substance use disorders are then viewed as a transition from voluntary drug use to compulsive drug-seeking habits^1^. Although this theory remains controversial^3^, there is some evidence that supports it. Drug addicts are impaired on gambling and reversal learning tasks^4–6^, and their behaviors are biased toward habitual (as opposed to goal-directed) control^7–10^. In rodent models, cocaine exposure or self-administration results in cognitive inflexibility in reversal learning tasks^11,12^ and exposure to various drugs of abuse, including cocaine^13,14^, ethanol^15–17^, methamphetamine^18,19^, and nicotine^20,21^ promotes the formation of habits, as evidenced by response insensitivity to outcome devaluation and/or contingency degradation. Finally, several studies have demonstrated persistent drug seeking despite punishment in rodents^22,23^.

However, the hypothesis that addiction is a compulsive drug-seeking habit is difficult to reconcile with other evidence, notably those showing that drug addicts remain remarkably sensitive to alternative incentives in the environment, suggesting flexible and goal-directed decision-making. For instance, rewarding abstinence with voucher in contingency management programs has proven effective in promoting long-term abstinence from cocaine^24,25^. Furthermore, a growing number of studies in rodents are showing that providing an alternative nondrug reward (e.g., sweet solution or food) during drug self-administration generally decreases drug intake. This finding is particularly true in studies involving mutually exclusive choice between cocaine and a palatable food reward, in absence of drug influence. In these studies, rats readily quit drug use to express a robust preference for the alternative nondrug reward^26–32^, even after having escalated their drug intake^27,32^. It was recently shown that rats also preferred social interaction over heroin and methamphetamine, demonstrating that preference for the nondrug alternative is not limited to gustatory rewards^33^. More generally, drug self-administration can be affected by numerous manipulations applied to the drug or to the alternative nondrug reward, such as price, dose or delay^26,27,34^. Together, these studies seem to indicate that contrary to the compulsive drug habit hypothesis, rats would remain able to allocate, apparently flexibly, their choice according to the value of the available options.

However, this conclusion presupposes that rats’ preference for the nondrug option mainly results from flexible goal-directed decision-making, though this assumption has so far not been directly demonstrated. Indeed, in most choice studies, rats were trained to respond for each reward separately before choice testing. In these conditions, one cannot exclude that rats’ subsequent preference for the nondrug option is the result of a previously established rigid habitual decision-making process. As explained above, we know that prior drug use can promote habit formation in rats. However, whether the resulting habits are inflexible or not is currently largely unknown. This is mainly due to the fact that rats’ sensitivity to outcome devaluation is tested under extinction, notably to avoid that direct experience of devaluation during testing prompts a switch back to goal-directed control^35^. However, if habitual decision-making has also become inflexible, as hypothesized here, then such a switch should not occur and, thus, even reinforced responding should be insensitive to devaluation, at least initially. The objective of this study was to directly address this question, by testing rats’ sensitivity to the current value of the nondrug option. We selected water as the alternative nondrug reward mainly because its value can be manipulated relatively easily through water-restriction and satiation, without any confounding hedonic component. In theory, if rats’ decision-making is both habitual and inflexible (i.e., unable to switch back to a goal-directed mode upon experiencing a change in options value), their preference should be largely insensitive to a sudden change in the preferred option value (i.e., devaluation induced by water satiety). On the contrary, their preference could only change after repeated training with the novel value.

## Materials and methods

### Animals

A total of 28 male Wistar rats (Charles River, L’Arbresle, France) weighing in average 315 g at the start of the experiment were housed in groups of 2 and maintained in a light- (reverse light–dark cycle) and temperature-controlled vivarium (22–23 °C). All behavioral testing occurred during the dark phase of the light–dark cycle. Home-cages were enriched with a nylon gnawing bone and a cardboard tunnel (Plexx BV, The Netherlands). Food was freely available in the home-cages throughout the duration of the experiment. Under water restriction, rats had 1 h of water access per day immediately after devaluation sessions, and 2 h after regular sessions. Body weight and water intake were monitored daily throughout the experiment. Three rats were lost during the experiment because of failed catheter patency (*n* = 2) or a failure to meet the criteria for acquisition of operant behavior (*n* = 1), thereby leaving 25 rats for final analysis.

### Ethical statement

All experiments were carried out in accordance with institutional and international standards of care and use of laboratory animals [UK Animals (Scientific Procedures) Act, 1986; and associated guidelines; the European Communities Council Directive (2010/63/UE, 22 September 2010) and the French Directives concerning the use of laboratory animals (décret 2013–118, 1 February 2013)]. The animal facility has been approved by the Committee of the Veterinary Services Gironde, agreement number A33–063–922.

### Surgery

Rats were surgically prepared with an indwelling silastic catheter in the right jugular vein under deep anesthesia. Behavioral testing commenced at least 7 days after surgery.

### General behavioral procedures

#### Initial operant training

**Fig. 1:**
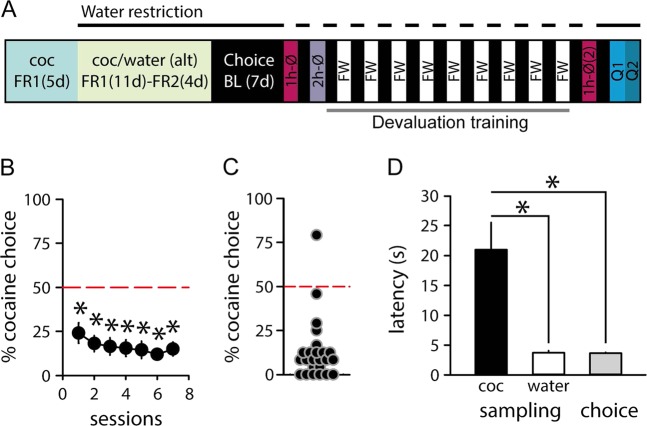
The choice between water and cocaine under water restriction. **a** Experimental timeline. **b** Mean percent of cocaine choices (±SEM) as a function of testing sessions. The horizontal dashed line at 50% represents the indifference level. * Different from the indifference level (*p* < 0.001, *t*-test). **c** Distribution of individual preference scores averaged over the last three choice sessions. **d** Mean latency (±SEM) to select the cocaine or water option during sampling or choice trials. *Different from cocaine sampling latency (*p* < 0.001)

Before any behavioral testing, rats were first trained to self-administer cocaine for 5 sessions under a fixed-ratio 1 (FR) schedule of reinforcement. Next, rats were water restricted and trained for 2 weeks under a FR1 (11 sessions) and then FR2 (4 sessions) schedule of water and drug self-administration on alternating daily sessions, six sessions a week (Fig. 1a). On water sessions, completion of the ratio on the lever located in the middle of the left wall of the chamber, was rewarded by a 20-s access to discrete volumes (0.04 ml) of water delivered in the adjacent drinking cup and was signaled by illumination of the cue-light above the lever. The first 2 volumes were delivered freely during the first 3 s to fill the drinking cup; subsequent volumes were obtained by licking (1 volume per 20 licks in about 2.8 s). On drug sessions, completion of the FR requirement on the lever located in the middle of the right wall of the chamber, was rewarded by one intravenous dose of 0.25 mg cocaine and was signaled by illumination of the cue-light above the drug lever. Water and drug sessions ended after rats had earned a maximum of 20 rewards or 2 h had elapsed.

#### Choice procedure

Each daily choice session consisted of 12 discrete trials spaced 10 min apart and divided into two successive phases: reward sampling (4 trials) and choice (8 trials). Sampling trials started with the presentation of one lever at a time in the following order: drug lever–water lever–drug lever–water lever. Two consecutive lever-presses within 5 min on the available lever were rewarded with the corresponding reward. Reward delivery was signaled by retraction of the lever and illumination of the cue light above the lever. Failure to respond within 5 min resulted in lever retraction with no cue-light nor reward delivered. During the choice phase, each trial started with the simultaneous presentation of water and drug levers. Rats were allowed to respond on either of these two levers to self-administer the corresponding reward. Reward delivery was signaled by retraction of both levers and illumination of the cue-light above the selected lever. If rats failed to respond to either lever within 5 min, the trial was considered as an omission, both levers retracted and no reward was delivered. Importantly, a 10-min inter-trial interval (ITI) was used here to prevent the acute effects of the cocaine administered on a given trial to influence the outcome of the choice on the next trial^27,36^.

### Behavioral experiment

Rats were first tested in the choice procedure under water restriction to establish baseline preference (Fig. 1a). To induce satiety, rats were given 1- then 2-h water access in their home-cage, immediately before the choice session (conditions 1 h-Ø and 2 h-Ø, separated by one baseline session under water deprivation). Water bottles were returned to the home-cages for 1 h immediately after these sessions to assess satiation.

To ensure a maximal possible level of satiation during choice sessions, water was made available for 1 h immediately before the choice session and during every inter-trial intervals (ITI) of the session. More specifically, discrete volumes of water (0.04 ml) were freely delivered “on demand” by licking in the drinking cup during ITIs (1 volume per 20 licks in about 2.8 s) but not during trials, unless rats selected the water option. Satiation was assessed by allowing rats to drink water for 1 h in their home-cage immediately after each session. These “free water” (FW) sessions were conducted in alternation with deprivation sessions for 9 cycles. During this experimental phase (“devaluation training”), rats progressively learned to reverse their preference in favor of cocaine during FW sessions. To investigate the learning mechanisms underlying preference reversal, rats were tested again in the 1 h-Ø condition with 1-h water access before the session, but no water deliveries during ITIs. After one baseline session under deprivation, thirsty rats were given choices between cocaine and water adulterated with 0.05% quinine across 2 consecutive sessions (Q1 and Q2, Fig. 1a).

### Drugs

Cocaine hydrochloride (Coopération Pharmaceutique Française, Melun, France) was dissolved in 0.9% NaCl, filtered through a syringe filter (0.22 µm) and stored at room temperature. Drug doses are expressed as the weight of the salt.

### Analyses

The percentage of water self-delivered upon licking during each 17-s water access was used as an index of satiation and was computed as follow; 100*volume self-administered/ total volume available (0.24 ml maximum per 17-s water access). Test sessions were compared to the preceding deprivation session (baseline). All data were subjected to relevant repeated-measures ANOVAs, followed by Newman–Keuls post-hoc tests where relevant. Comparisons with a fixed theoretical level (e.g., 0 or 50%) were conducted using one sample *t*-tests. Some behavioral variables had no variance and were thus analyzed using non-parametric statistics (i.e., Friedman’s test for the main effect followed by Wilcoxon’s test for paired comparisons). Repeated-measures of nominal data were analyzed with the McNemar’s test. A significant level of *p* < 0.05 was used for all statistical analyses. Statistical analyses were run using Statistica, version 7.1 (Statsoft Inc., Maisons-Alfort, France).

## Results

Under water restriction, rats expressed a strong preference for water over cocaine from the first choice session (Fig. 1b). Indeed, 96% of rats preferred water with only one out of 25 rats preferring cocaine (Fig. 1c). In accordance with their preference, rats responded faster for water than cocaine during sampling trials (*t*_(25)_ = 3.77, *p* < 0.001) and rapidly selected the water option during choice trials (Fig. 1d).

**Fig. 2:**
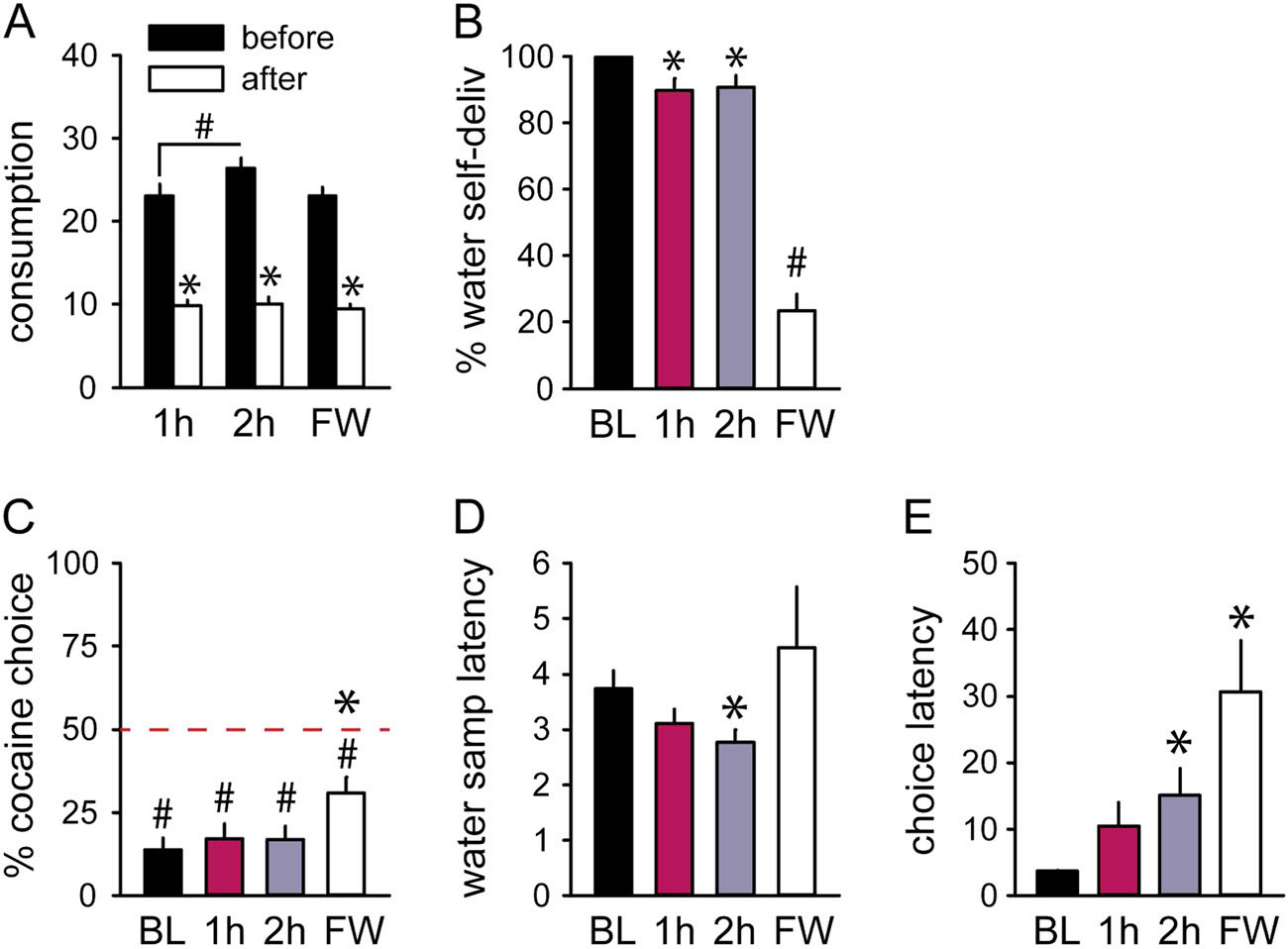
Partial effect of satiation on water preference in 1 h-Ø (1 h), 2 h-Ø (2 h) and Free Water (FW) sessions. **a** Mean water consumption in mL (±SEM) during home-cage water accesses before (black bars) and after (white bars) choice sessions. *Different from before (*p* < 0.0001). # 2 h different from 1 h (*p* < 0.001). **b** Suppression of water intake, expressed in percent of volume self-delivered (±SEM) per total volume available during water trials. *Different from baseline (*p* < 0.01). ^#^Different from baseline (*p* < 0.0001). **c** Mean percent of cocaine choices (± SEM) under conditions of deprivation and satiation. The horizontal dashed line at 50% represents the indifference level. *Different from baseline (*p* < 0.0001). ^#^Different from indifference (*p* < 0.001). **d** Mean water sampling latency (±SEM) under conditions of deprivation and satiation. *Different from baseline (*p* < 0.05). **e** Mean choice latency (±SEM) under conditions of deprivation and satiation. *Different from baseline (*p* < 0.01)

1 or 2 h water access immediately before the choice session induced satiation, as indicated by the reduction of water consumption in the home-cage after the session compared to before (Fig. 2a) (*F*_(1,13)_ = 79.61, *p* < 0.0001). Increasing the duration of water access to 2 h did not increase satiation since water intake was reduced to a similar extent as the 1 h-Ø condition (Fig. 2a) (*p*-value > 0.05). These manipulations resulted in a minor but significant suppression of water intake during water trials (Fig. 2b and S1) (Friedman Chi-Square = 22.58, *p* < 0.0001). However, pre-session water access had no effect on preference (Fig. 2c) (*F*-value < 0.8, *p*-value > 0.05) and a marginal effect on water sampling latency (Fig. 2d) (water sampling latency: *F*_(2,48)_ = 3.97, *p* < 0.05). Choice latency was however increased after pre-session water accesses (session: *F*_(2,48)_ = 5.57, *p* < 0.01) with a longer latency in the 2 h-Ø condition relative to baseline (*p* < 0.01) (Fig. 2e).

These results suggest that 1 h-Ø and 2 h-Ø conditions might not have produced enough satiation to affect preference. To ensure a maximal possible level of satiation during the session, water was also made available during every ITIs of the session (“Free Water”—FW condition). On average, rats consumed 6.5 ± 0.8 mL of water during this FW session, mainly during the first ITI (Fig. S2). Analysis of home-cage water consumption before and after the FW session reveals that satiation was comparable with the previous 1 h- and 2 h-Ø conditions (*F*-values < 0.2, *p*-values > 0.05) (Fig. 2a). However, in marked contrast to these conditions, water consumption was drastically suppressed during water trials (Fig. 2b and S1) (*Z*_(25)_ = 4.37, *p* < 0.0001 against baseline). Although preference for cocaine slightly increased compared to baseline (Fig. 2c) (*t*_(24)_ = 4.85, *p* < 0.0001), it remained significantly below indifference level (*t*_(24)_ = 4.51, *p* < 0.001). The latency to sample water was not significantly altered during the FW session (Fig. 2d) (*t*-value < 1.6, *p* > 0.05) but rats were slower to select an option during choice trials (Fig. 2e) (*t*_(24)_ = 3.58, *p* < 0.01).

**Fig. 3:**
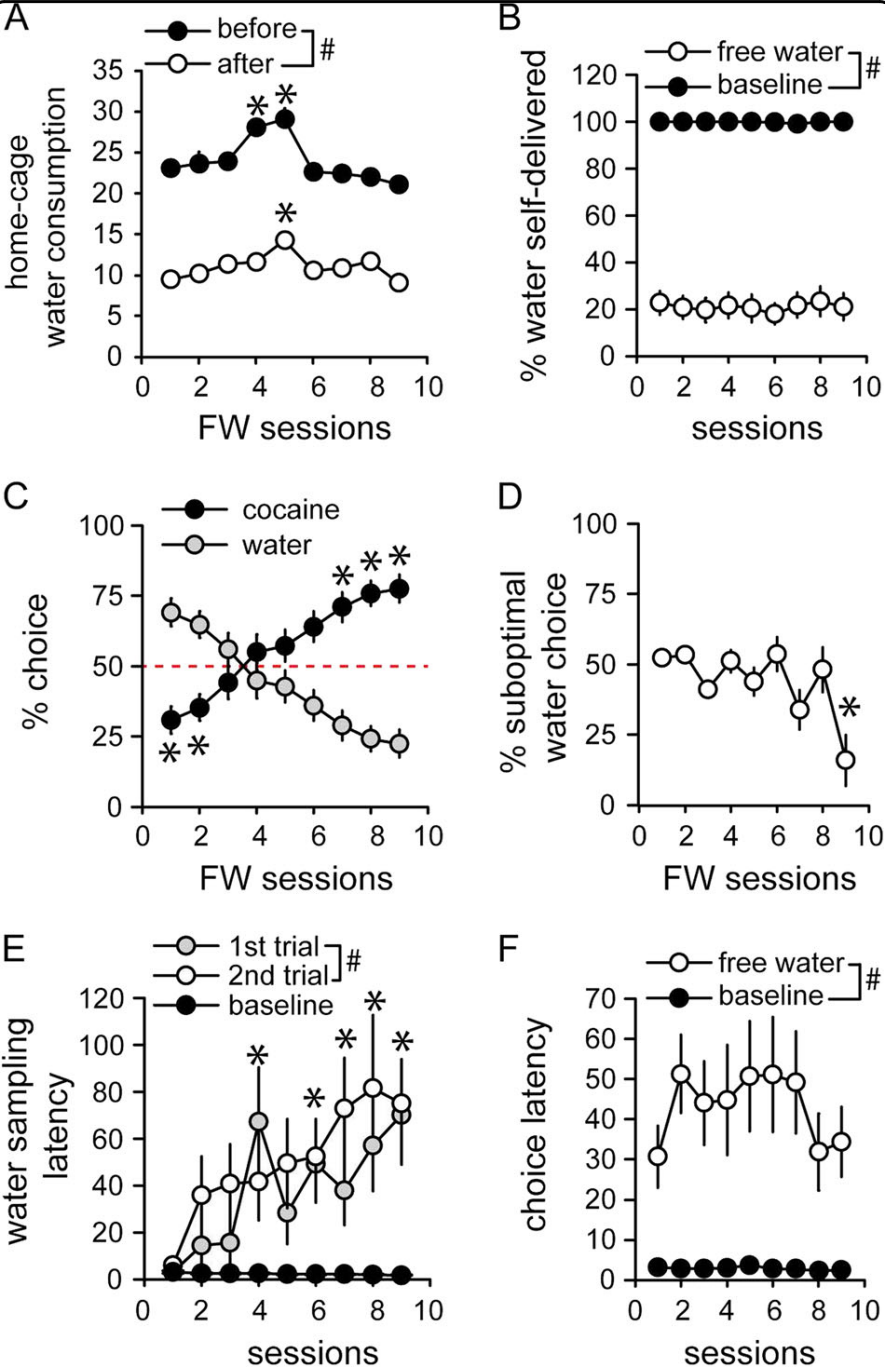
Slow reversal of preference during devaluation training. **a** Mean water consumption in mL (±SEM) during home-cage water accesses before (black circles) and after (white circles) Free Water (FW) sessions. ^#^Difference between before and after (*p* < 0.0001). *Different from the first FW session (*p* < 0.001). **b** Percent of volume self-delivered (±SEM) across deprivation (baseline) and FW sessions. ^#^Difference between baseline and FW sessions (*p* < 0.0001). **c** Mean percent of cocaine and water choices (±SEM) as a function of FW sessions. The horizontal dashed line at 50% represents the indifference level. *Different from the indifference level (*p* < 0.05, *t*-test). **d** Mean percent of suboptimal water choices not followed by consumption (±SEM), as a function of FW sessions. *Different from first FW session (*p* < 0.01). **e** Mean water sampling latency (±SEM) in the first and second trials of FW sessions (gray and white circles) and during baseline sessions (black circles). ^#^Difference between 1st and 2nd sampling trials of FW sessions (*p* < 0.05). *Different from baseline sessions (*p* < 0.05). **f** Mean choice latency (±SEM) across FW and baseline sessions. ^#^Difference between Free Water and baseline sessions (*p* < 0.0001)

Since the FW session was accompanied by a slight, albeit significant effect on preference, FW sessions were repeated in alternation with deprivation sessions for 9 cycles (Fig. 1a and S3). Water intake before and after each FW sessions was stable with no increasing or decreasing trend across sessions (Fig. 3a) (session *F*_(8,104)_ = 21.28, *p* < 0.0001; first vs last FW session *p* > 0.05). Despite steady suppression of within-session water intake across FW sessions (Friedman Chi-square = 363.5, *p* < 0.0001) (Fig. 3b and S1), the change in preference was gradual (Fig. 3c and S3) (*F*_(8,192)_ = 10,01, *p* < 0.0001). On average, preference for cocaine over water appeared on the fourth session but differed significantly from indifference on the 7th FW session (Fig. 3c and S3). Persistence of water choice despite satiation is surprising. In fact, around 50% of water choices were not followed by water consumption during the first 6 FW sessions (Fig. 3d). This percentage then declined to reach 16% on the last FW session (Fig. 3d) (session: *F*_(8,192)_ = 3.42, *p* < 0.01). The reduction of water choices during FW sessions was correlated with a progressive increase in water sampling latency (Fig. 3e and S4) (session: *F*_(8,192)_ = 3.82, *p* < 0.001; satiation: *F*_(1,24)_ = 23.08, *p* < 0.0001, satiation by session interaction *F*_(8,192)_ = 4.06, *p* < 0.001). Interestingly, water sampling latency was longer on the second trial compared to the first (Fig. 3e) (*F*_(1,24)_ = 7.35, *p* < 0.05). Choice latency was higher in FW sessions relative to deprivation sessions (*F*_(1;24)_ = 22.66, *p* < 0.0001), but did not differ across sessions (Fig. 3f). Importantly, persistent water choice during the first FW sessions did not result from a bias in preference in favor of water. Indeed, comparable results were obtained when comparing responding for water during sampling trials against its own baseline on the preceding privation session (Fig. S5).

**Fig. 4:**
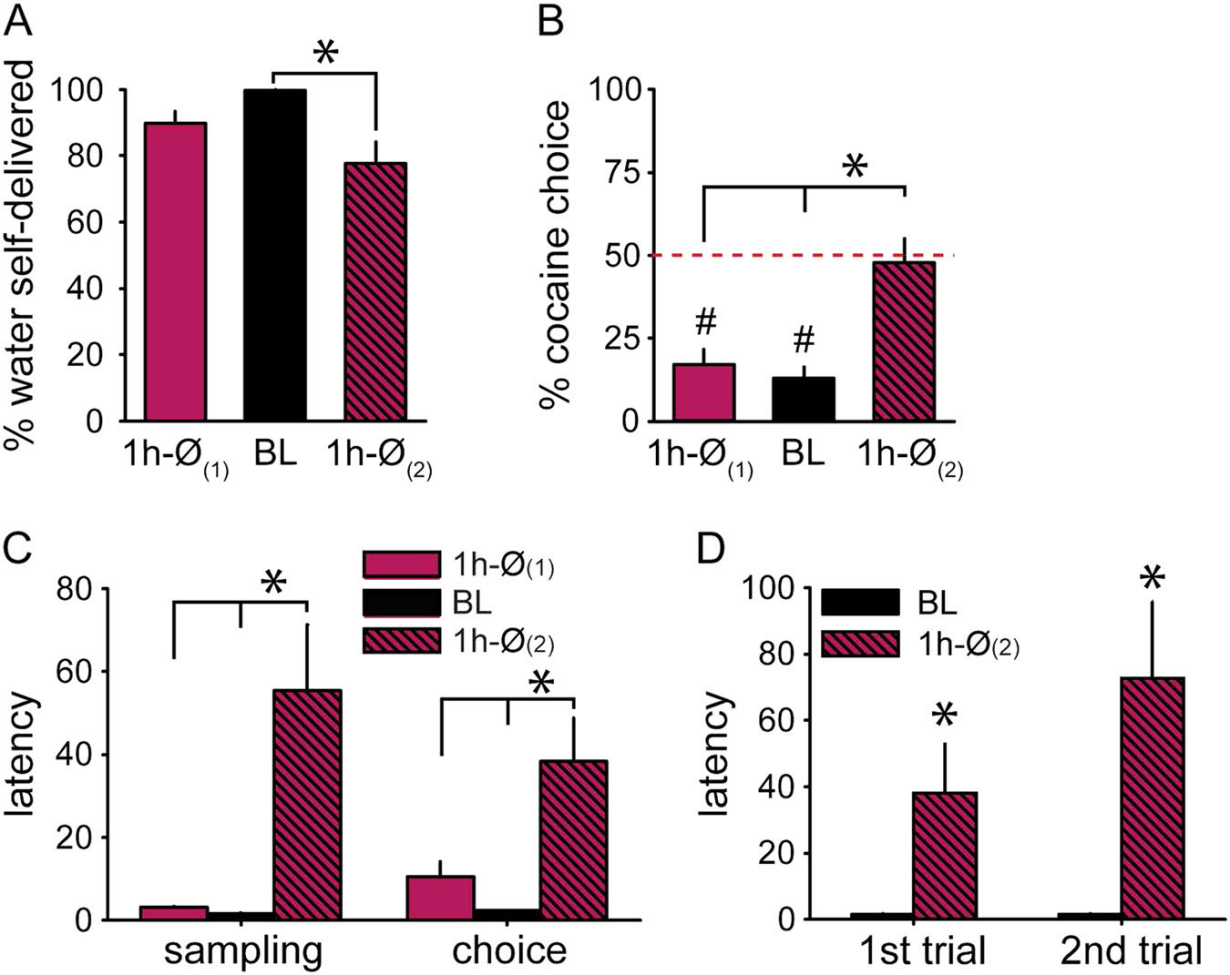
Higher sensitivity to devaluation by satiation in the 1 h-Ø session after devaluation training. **a** Percent volume of water self-delivered (±SEM) during 1 h-Ø_(1)_, baseline, and 1 h-Ø_(2)_ sessions. *Different from 1 h-Ø_(2)_ (*p* < 0.0001). **b** Mean percent of cocaine choices during 1 h-Ø_(1)_, deprivation, and 1 h-Ø_(2)_ sessions. The horizontal dashed line at 50% indicates the indifference level. *Different from 1 h-Ø_(2)_ (*p* < 0.0001). ^#^Different from indifference (*p* < 0.0001). **c** Mean water sampling and choice latency (±SEM) during 1 h-Ø_(1)_, baseline, and 1 h-Ø_(2)_ sessions. *Different from 1 h-Ø_(2)_ (*p* < 0.01). **d** Mean water sampling latency (±SEM) in the first and second trials of baseline and 1h-Ø_(2)_ sessions. *Different from baseline (*p* < 0.001)

Following devaluation training, rats were tested again in the 1h-Ø condition, with 1 h water access before the session, but no water access during the ITIs (1 h-Ø_(2)_ session). Water consumption was significantly suppressed in the 1 h-Ø_(2)_ session compared to baseline (Fig. 4a and S1) (*Z*_(25)_ = 4.11, *p* < 0.0001). Preference was sensitive to devaluation in the 1 h-Ø_(2)_ session, as indicated by higher percentage of cocaine choice (Fig. 4b) (*t*_(24)_ = 5.82, *p* < 0.0001) and longer water sampling and choice latencies compared to baseline (Fig. 4c) (water sampling: *t*_(24)_ = 3.39, *p* < 0.01, choice: *t*_(24)_ = 3.45, *p* < 0.01). Water sampling latency was increased from the first trial, suggesting that rats updated the water option value based on their motivational state at the start of the session (Fig. 4d) (*F*_(1,24)_ = 11.51, *p* < 0.01). Interestingly, the 1h-Ø condition after devaluation training (1 h-Ø_(2)_) produced higher effects on preference (Fig. 4b) (*t*_(24)_ = 4.69, *p* < 0.0001) and latencies (Fig. 4c) (sampling: *t*_(24)_ = 3.31, *p* < 0.01; choice: *t*_(24)_ = 2.67, *p* < 0.05) than the same 1 h-Ø condition before devaluation training (1 h-Ø_(1)_). These differences suggest that during devaluation training, rats have learned to update the value of the water option based on their motivational state at the start of the session. Alternatively, it is possible that rats progressively learned to flexibly adjust their responding to the devalued reward. To assess this possibility, rats were required to choose between cocaine and water adulterated with 0.05% quinine, under water restriction. If rats had learned to compute the water option value based on their motivational state, then one would expect a preference for water over cocaine. On the contrary, if devaluation training allowed rats to restore flexible, goal-directed decision-making, then one would expect rats to be sensitive to water adulteration and reverse their preference in favor of cocaine.

**Fig. 5:**
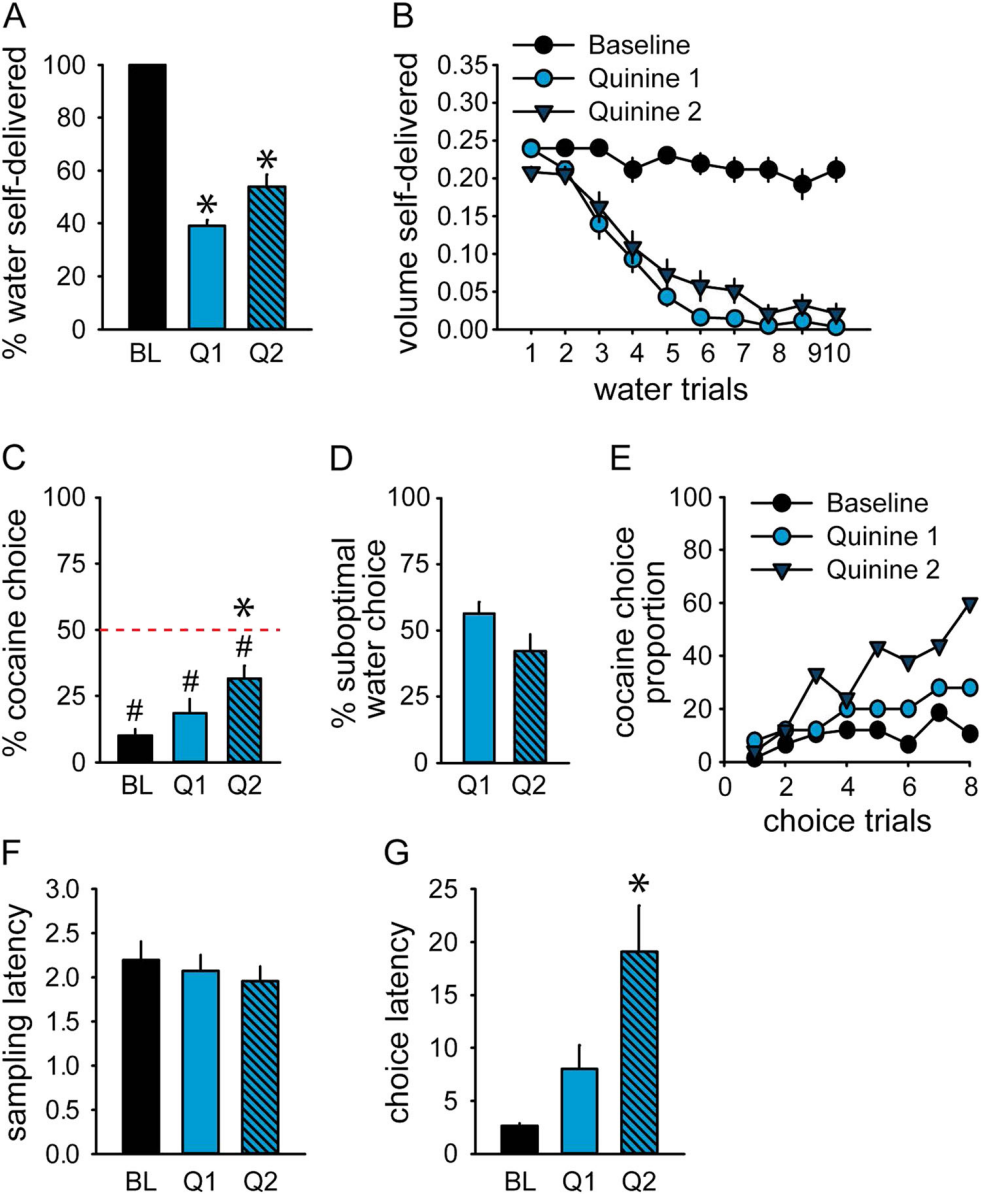
Low preference sensitivity to devaluation of water with 0.05% quinine. **a** Percent volume of water self-delivered (±SEM) during baseline (BL), quinine 1 (Q1) and quinine 2 (Q2) sessions. *Different from baseline (*p* < 0.0001). **b** Mean volume of water self-delivered (±SEM) across water trials during baseline, quinine 1 and quinine 2 sessions. **c** Mean percent of cocaine choices during baseline, quinine 1 and quinine 2 sessions. The horizontal dashed line at 50% indicates the indifference level. *Different from deprivation (*p* < 0.001). ^#^Different from the indifference level (*p* < 0.001). **d** Mean percent of suboptimal water choices not followed by consumption (±SEM) in quinine 1 and quinine 2 sessions. **e** Proportion of rats selecting the cocaine option across choice trial during baseline, quinine 1 and quinine 2 sessions. **f**, **g** Mean water sampling (**f**) and choice (**g**) latency (±SEM) during baseline, quinine 1 and quinine 2 sessions. *Different from baseline (*p* < 0.001)

Adulteration of water with 0.05% quinine significantly reduced water intake in the two quinine sessions (Q1 and Q2) compared to baseline (Fig. 5a and S1) (Friedman Chi-Square = 38.02, *p* < 0.0001). However, trial-by-trial analysis of water self-administration reveals that on the first quinine session, water intake was significantly suppressed from the second sampling trial (all *Z*-values > 2.8, *p*-values < 0.01) and keep decreasing over trials until a complete suppression on trials 8 to 10 (Fig. 5b). Water intake followed the same time-course on the second quinine session with a significant suppression from the first sampling trial (Fig. 5b). Although the percentage of cocaine choice increased compared to baseline (Fig. 5c) (session, *F*_(2,48)_ = 8.11, *p* < 0.001), it only reached significance on the second quinine session (*p* < 0.001) and remained below indifference level (Q1: *t*_(24)_ = 5.99, *p* < 0.0001; Q2: *t*_(24)_ = 4.00, *p* < 0.001). In fact, about 50% of water choices were not followed by water consumption, which illustrates the inflexible nature of rats’ decision-making under this condition (Fig. 5d). The proportion of rats selecting the cocaine option slightly increased across trials on Q1 and Q2, suggesting that experiencing the devalued reward partially promoted cocaine choice (Fig. 5e) (McNemar Chi-square trial 1 vs 10; Q1: 10.32, *p* < 0.01; Q2: 7.11, *p* < 0.01). Although water sampling latency did not differ from baseline (Fig. 5f) (*F*-value < 0.5, *p*-value > 0.05), choice latency increased across sessions (*F*_(2,48)_ = 9.93, *p* < 0.001) and significantly differed from baseline on Q2 (Fig. 5g) (*P* < 0.001). The difference in preference (*p* < 0.05) and choice latency (*p* < 0.01) between sessions Q1 and Q2 suggests that a new learning is occurring.

## Discussion

We have previously shown that reward palatability can surpass cocaine reward^27^. Here, we are extending this result to a non-palatable biological reward by showing that under water restriction, rats robustly preferred water over cocaine. This indicates that satisfaction of a primary biological need (i.e., thirst) can be more reinforcing than cocaine. Overall, water preference was insensitive to sudden water devaluation. Only with repeated devaluation training did we observe a progressive reversal of preference toward the drug. Additional tests showed that this preference reversal did not result from the restoration of goal-directed control but resulted instead from a new interoceptive discrimination learning. Previous research showed rapid habit formation after prior exposure to cocaine. The present study confirms and extends this research by showing that once formed, habits are also inflexible. Some of the possible mechanisms and factors that contribute to this inflexibility are discussed below. Finally, the present study also shows that inflexible decision-making does not necessarily promote drug choice, but can also under some circumstances favor drug abstinence.

Persistent responding for water despite its devaluation by satiation in FW sessions demonstrates that choice of the alternative nondrug reward is under habitual control. Although water consumption was suppressed by 80%, rats kept selecting the water option on 100% of sampling trials and 65% of choice trials on the first FW session. Persistent water choices could not be explained by the fact that though devalued, water remained nonetheless more valuable than cocaine. Indeed, about 50% of water choices were not followed by water consumption, which resulted in high opportunity cost by preventing cocaine self-administration. Furthermore, rats eventually shifted their choice to cocaine during devaluation training (with 88% of rats expressing a preference for cocaine during the last FW session), demonstrating that, in the satiated state, water had indeed become less valuable than cocaine. Beside its satiating effect, free water access during ITIs also participates to degrade the contingency between responding and water deliveries in the FW condition. This manipulation is also expected to suppress goal-directed but not habitual responding^37,38^, further suggesting that water choice is under habitual control in our conditions.

Although water preference was initially resistant to this combination of water devaluation and contingency degradation, rats eventually learned to reverse their preference, in favor of cocaine with extended devaluation training. This progressive reversal of preference from the devalued to the non-devalued option is what should be expected under habitual control, as hypothesized here. Indeed, one would expect an immediate reversal of preference under goal-directed control since rats were given the opportunity to directly experience the devalued outcome before choice. Direct experience with water in the novel sated state should have, in theory, updated the internal representation of water value and prompted a change in preference toward the non-devalued option^35^. It could be argued that goal-directed and habitual control are engaged in parallel during choice sessions, and that progressive dominance of goal-directed over habitual system would account for the progressive reversal observed in this study^39^. If this was true, preference would be under complete goal-directed control by the end of devaluation training, and a preference for cocaine would then be expected following quinine-induced devaluation. Yet, rats maintained their preference for water on both quinine sessions.

These results rather suggest that rats’ behavior was under habitual control, and that the value of the water option was iteratively and retrospectively updated with repeated experience of water under deprivation and satiation^39^. Such learning is generally slow and requires repeated experience. To select the best option, rats may have learned to compute actions’ value based on their motivational state (i.e., select water when thirsty, and cocaine when sated), without relying on the expected current value of these two rewards. Since quinine sessions were conducted under deprivation, this hypothesis can explain why rats kept choosing water despite adulteration with quinine. Learning to use motivational state as an interoceptive cue to predict the most valuable outcome could also explain why testing rats in the 1 h-Ø condition after devaluation training was sufficient to increase preference for cocaine to 50% whereas the same condition had no effect on preference before devaluation training. It is worth pointing out that alternation of choice sessions under deprivation and satiation during devaluation training may have promoted this state-dependent decision-making. Further research is necessary to investigate changes in rats’ preference under continuous conditions of outcome devaluation.

Analyses of sampling latencies also support the hypothesis of state-dependent learning. While rats were very fast to sample water on deprivation sessions, response latencies progressively became longer during FW sessions, and the time-course of latency lengthening was nicely correlated with the shift in preference toward cocaine. Thus, as previously suggested^40^, response latency during water sampling trials can constitute a reliable index of the water option value. Importantly, water sampling latencies were increased from the first sampling trial, even during the 1 h-Ø_(2)_ session, in which water cannot be experienced during ITIs. This suggests that motivational state at the start of the session was sufficient for rats to predict the water option value. Yet, this option value could also be partially updated within-session through consumption of water during trials or ITIs, as indicated by the longer response latency on the second water sampling trial relative to the first. This mechanism could also explain within- and between-session increases in cocaine choice across the 2 quinine sessions.

Together, these results suggest that preference for the nondrug option could result from previously established habitual decision-making. This inflexible decision-making may have resulted from prior cocaine self-administration, which was nevertheless relatively modest in the present study (i.e., 10 2-h sessions). Indeed, previous research has shown that cocaine exposure or self-administration produces numerous alterations in corticostriatal circuits involved in behavioral control^41,42^. Besides its well-known facilitating effects on habit formation^13,14^, cocaine exposure also impairs cognitive flexibility in rodents and monkeys in reversal learning tasks^11,12,43^. Furthermore, cocaine seeking can persist despite punishment after escalation of cocaine self-administration^22,23,44^. Together, these studies suggest that in our experiment, prior cocaine exposure may have compromised rats’ ability to flexibly engage goal-directed control, in order to guide their choice in the face of changing circumstances. Strong habit formation, impaired goal-directed control or dysfunctional arbitration between these 2 systems could underlie the inflexibility reported here^45–47^. However, the presumed role of cocaine in this inflexibility must be taken with caution since no control group or condition for cocaine exposure was included in the present study. In fact, it is not clear how this control can be implemented in a relevant manner when the primary dependent variable is cocaine choices.

The present results appear to contradict previous studies showing that extended drug self-administration is necessary to drive habitual learning^48^ (but see refs. ^49,50^) and that expression of habit is prevented in tests involving a choice between multiple response-outcome associations^51,52^. However, the procedures used here differed from previous studies in two significant ways. First, we used a discrete-trials procedure as opposed to free-operant schedules generally used to assess behavioral control. It was shown that rats trained in a discrete-trial procedure are prone to rapidly develop habit^53^, an effect presumably mediated by higher reinforcer predictability^54^. Secondly, we investigated sensitivity to outcome devaluation and contingency degradation in repeated reinforced choice sessions, in which rats can directly experience the devalued reward. To our knowledge, this study is the first to investigate habitual control in such an experimental setting. Further research under normal (nondrug) conditions is thus necessary to delineate the relative contribution of cocaine exposure in the inflexible decision-making reported here.

It could be argued that the strong baseline difference in preference for cocaine and water may prevent the meaningful assessment of sensitivity to outcome devaluation. Indeed, in devaluation sessions, a decrease in responding for water could have been masked by the high preference for water under baseline condition. However, this is unlikely since the initial insensitivity to water devaluation was also observed in absence of choice, during water sampling trials (Fig. S5). More specifically, responding for water during sampling trial was not reduced compared to baseline during the sessions 1 h-Ø, 2 h-Ø and the first FW-session. However, a devaluation effect appeared on subsequent FW sessions.

Several studies suggest that habitual behavior develops more readily for drugs of abuse than for natural rewards^15,16^. Yet, the behavioral inflexibility evidenced in this study, presumably favored by prior exposure to cocaine, was directed at the alternative nondrug option. The strong reinforcing value of water in our conditions may have contributed to this finding. Indeed, drinking water is a biological need critical for survival that may partly explain the robust preference for water reported here, and its resistance to satiety-induced devaluation and quinine adulteration. Although paradoxical, these results are in agreement with previous studies showing that drug-induced habits are not specific to drug-seeking behavior per se and can affect responding for natural reward, more generally^15,18,19^. How these findings would generalize to another alternative nondrug reward such as saccharin, predominantly used in previous choice experiments from our laboratory, deserves additional research.

In conclusion, the present study holds with the development of cognitive inflexibility previously demonstrated after cocaine exposure or self-administration^55^, and shows that this inflexibility does not necessarily promote further drug use. In fact, as shown here and elsewhere^26,27,32^, inflexible decision-making seemed instead to favor the choice of nondrug alternatives and thus to protect rats from prolonged drug use. There is thus a clear dissociation between decision-making inflexibility and preference orientation. Whether such dissociation also exists in human addiction remains to be seen^3^. Additional research is necessary to define the conditions that make decision-making inflexibility conducive of the development of compulsive drug use^46^. Nevertheless, this study presents a comprehensive framework to better understand how inflexible decision-making can influence preference in a choice situation involving drug and nondrug rewards.

## Supplementary information

Supplemental
