# Supplementary material for: Inflexible habitual decision-making during choice between cocaine and a nondrug alternative
Source: Transl Psychiatry. 2019 Mar 6;9:109. doi: 10.1038/s41398-019-0445-2 (PMC6403316; doi:10.1038/s41398-019-0445-2)
Supplement: Supplementary file 1 — Supplemental [file 41398_2019_445_MOESM1_ESM.docx]

# **Inflexible habitual decision-making during choice between cocaine and a nondrug alternative**

Running title: **cocaine and inflexible choice**

**Vandaele Y.^1^, Vouillac-Mendoza C.^2,3^ & Ahmed S.H.^2,3^**

Supplemental online informations


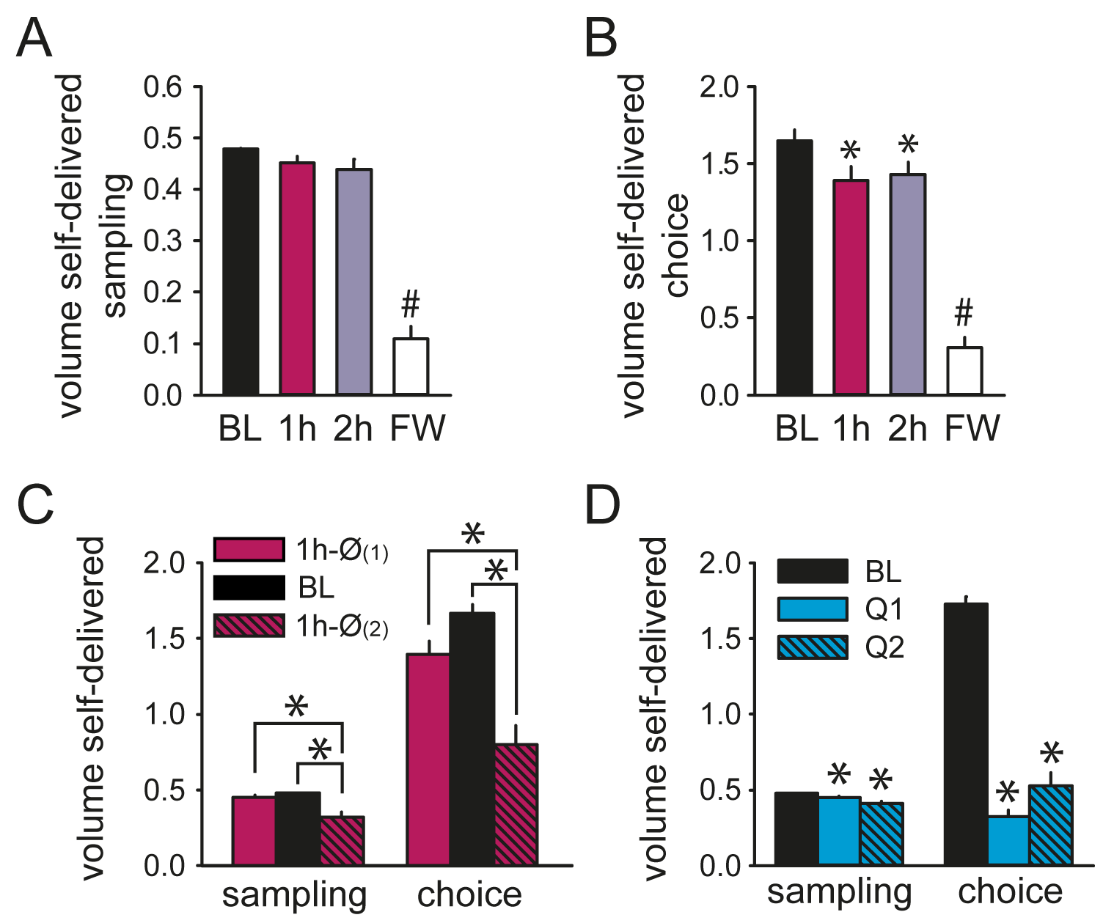


Figure S1: Water consumption during sampling and choice trials of test sessions. (A-B) Mean volume of water self-delivered in mL (±SEM) during sampling (A) and choice (B) trials under conditions of privation (baseline) and satiation in the 1h-Ø, 2h-Ø and free-water (FW) sessions. * Different from baseline (p<0.01). # Different from baseline (p<0.0001). (C) Mean volume of water self-delivered in mL (±SEM) during sampling and choice trials of the 1h-Ø_(2)_ session following devaluation training. *Different from baseline and 1h-Ø_(1)_ sessions (p<0.01). (D) Mean volume of water self-delivered in mL (±SEM) during sampling and choice trials of baseline, quinine 1 and quinine 2 sessions. *Different from baseline (p<0.001).


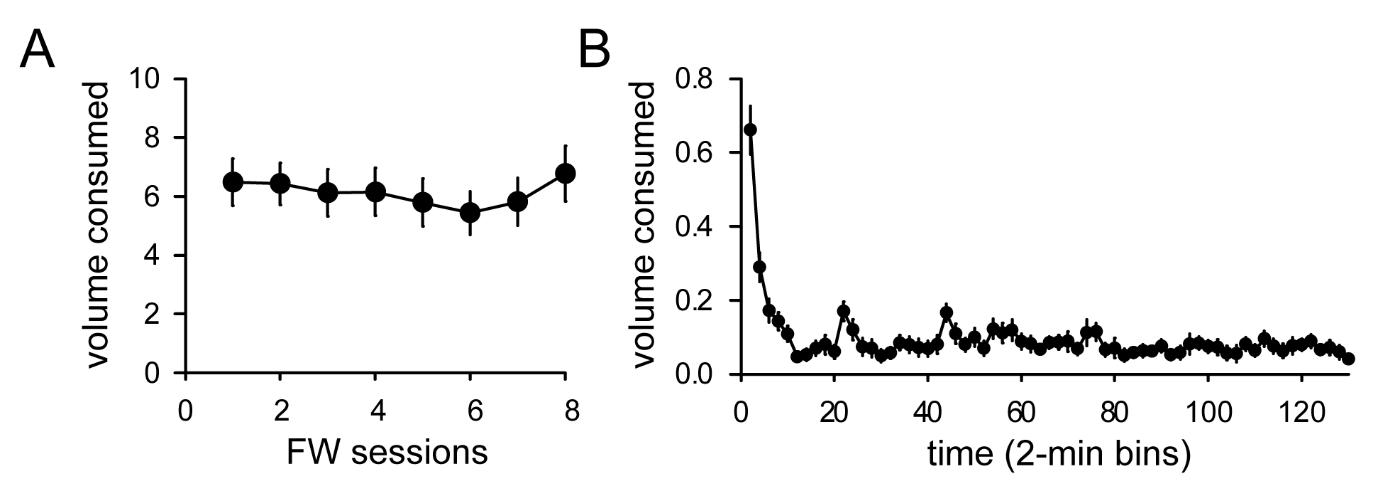


Figure S2: water consumption during ITIs of Free Water sessions. (A) Mean volume of water consumed (±SEM) during ITIs, as a function of Free Water sessions. (B) Mean (±SEM) within-session time-course of water consumption during continuous access to water during ITIs, averaged across the last three FW sessions.


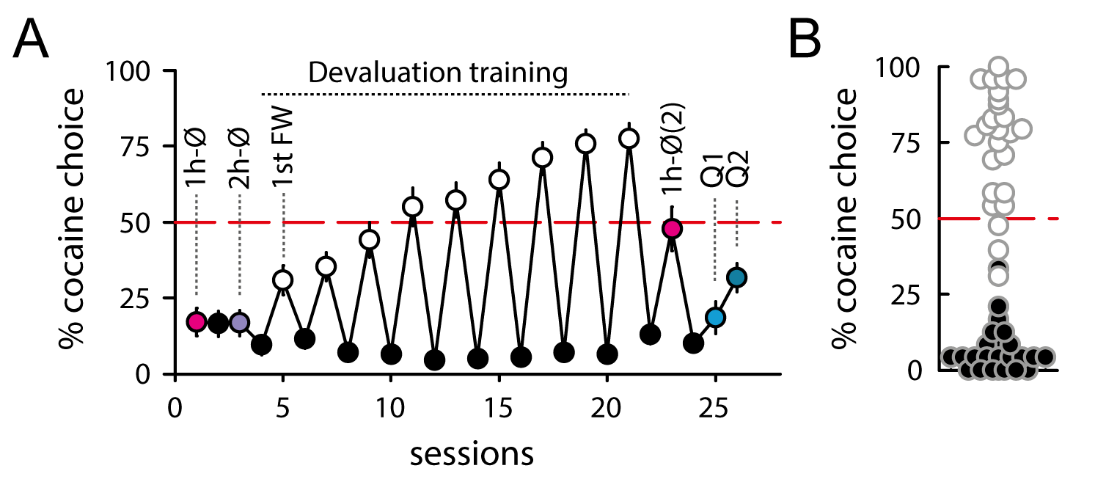


Figure S3. State-dependency of cocaine preference. A. Mean percentage of cocaine choice (±SEM) across choice sessions conducted under water restriction (black circle) or satiation (pink, purple and white circle). The last 2 quinine sessions (Q1 and Q2, blue circle) were conducted under water restriction. B. Distribution of individual preference scores averaged over the last three privation (black circles) and FW sessions (white circles). The horizontal dashed line at 50% represents the indifference level.


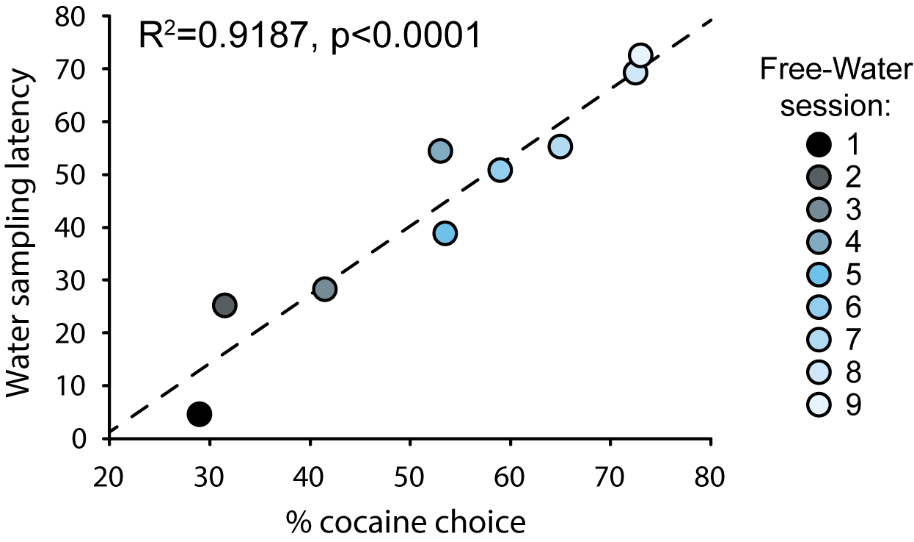


Figure S4: Pearson correlation between mean water sampling latency and mean preference for cocaine across FW sessions. The order of FW sessions is represented along a blue color gradient, with darker colors for earlier sessions. P<0.0001.


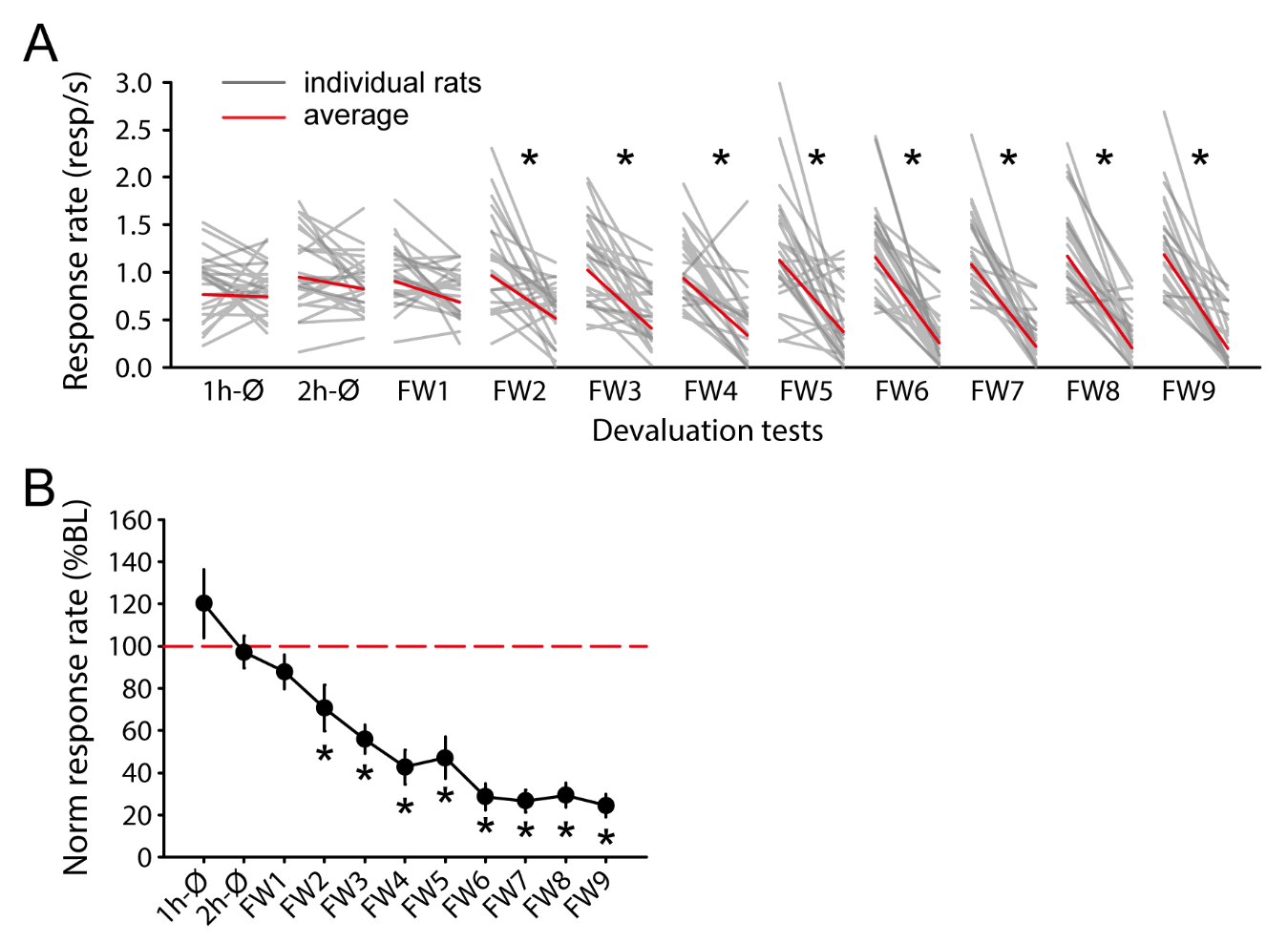


Figure S5: Assessment of the sensitivity to water devaluation during water sampling trials. A. Mean response rate during water sampling trials (in response per second) across devaluation testing. Gray lines indicate the response rate of individual rats during each satiety sessions in comparison to preceding baseline sessions. The red line indicates the average response rate. *p<0.05 compared to baseline B. Mean normalized response rate (in percentage of baseline responding) ±SEM. *p<0.05 against 100%.
